# The Osteocyte as the New Discovery of Therapeutic Options in Rare Bone Diseases

**DOI:** 10.3389/fendo.2020.00405

**Published:** 2020-07-08

**Authors:** Janak L. Pathak, Nathalie Bravenboer, Jenneke Klein-Nulend

**Affiliations:** ^1^Key Laboratory of Oral Medicine, Guangzhou Institute of Oral Disease, Affiliated Stomatology Hospital of Guangzhou Medical University, Guangzhou, China; ^2^Department of Clinical Chemistry, Amsterdam University Medical Centers, Amsterdam Movement Sciences, Vrije Universiteit Amsterdam, Amsterdam, Netherlands; ^3^Department of Oral Cell Biology, Academic Centre for Dentistry Amsterdam, Amsterdam Movement Sciences, University of Amsterdam and Vrije Universiteit Amsterdam, Amsterdam, Netherlands

**Keywords:** osteocyte, rare bone disease, mechanotransduction, bone remodeling, niche, sost/sclerostin, phosphate-homeostasis, RANKL

## Abstract

Osteocytes are the most abundant (~95%) cells in bone with the longest half-life (~25 years) in humans. In the past osteocytes have been regarded as vestigial cells in bone, since they are buried inside the tough bone matrix. However, during the last 30 years it has become clear that osteocytes are as important as bone forming osteoblasts and bone resorbing osteoclasts in maintaining bone homeostasis. The osteocyte cell body and dendritic processes reside in bone in a complex lacuno-canalicular system, which allows the direct networking of osteocytes to their neighboring osteocytes, osteoblasts, osteoclasts, bone marrow, blood vessels, and nerves. Mechanosensing of osteocytes translates the applied mechanical force on bone to cellular signaling and regulation of bone adaptation. The osteocyte lacuno-canalicular system is highly efficient in transferring external mechanical force on bone to the osteocyte cell body and dendritic processes via displacement of fluid in the lacuno-canalicular space. Osteocyte mechanotransduction regulates the formation and function of the osteoblasts and osteoclasts to maintain bone homeostasis. Osteocytes produce a variety of proteins and signaling molecules such as sclerostin, cathepsin K, Wnts, DKK1, DMP1, IGF1, and RANKL/OPG to regulate osteoblast and osteoclast activity. Various genetic abnormality-associated rare bone diseases are related to disrupted osteocyte functions, including sclerosteosis, van Buchem disease, hypophosphatemic rickets, and WNT1 and plastin3 mutation-related disorders. Meticulous studies during the last 15 years on disrupted osteocyte function in rare bone diseases guided for the development of various novel therapeutic agents to treat bone diseases. Studies on genetic, molecular, and cellular mechanisms of sclerosteosis and van Buchem disease revealed a role for sclerostin in bone homeostasis, which led to the development of the sclerostin antibody to treat osteoporosis and other bone degenerative diseases. The mechanism of many other rare bone diseases and the role of the osteocyte in the development of such conditions still needs to be investigated. In this review, we mainly discuss the knowledge obtained during the last 30 years on the role of the osteocyte in rare bone diseases. We speculate about future research directions to develop novel therapeutic drugs targeting osteocyte functions to treat both common and rare bone diseases.

## Introduction

Bone mainly contains three types of cells, i.e., osteocytes, osteoblasts, and osteoclasts. The osteocytes are the most abundant cells comprising 95% of the total cell population in bone with an average half-life of 25 years (1, 2). The bone-forming osteoblasts and bone-resorbing osteoclasts account for only ~5% of the total bone cell population, and live for only a few days to weeks. The characteristics and function of osteoblasts and osteoclasts during physiological bone remodeling and bone diseases have been extensively studied (3–6). However, the cellular and molecular mechanisms of osteocyte-mediated effects on skeletal health have not been fully elucidated. Five decades ago the osteocytes were still regarded as inert cells buried alive inside the bone matrix, despite the fact that the healthy human skeleton contains ~42 billion osteocytes (7). The mechanosensing property of osteocytes has been reported for the first time about three decades ago (8). With the advancement of new technologies in molecular and cellular mechanisms, imaging, transgenic approaches, and RNA sequencing, important functions of osteocytes and their role in bone homeostasis and vital systemic functions have become clear in the last two decades (1). Osteocytes are descendants of osteoblasts. During the bone mineralization process, some osteoblasts bury themselves in the bone matrix. They regulate mineralization, develop connective dendritic processes, and become osteocytes. Although osteocytes are buried deep inside the bone matrix, their dendritic processes are well-connected with neighboring osteocytes, osteoblasts, blood vessels, nerve cells, and bone marrow. The osteocyte cell body resides in a lacunar space inside the bone matrix. From the cell body 50–60 dendritic processes radiate in canaliculi space, forming a complex osteocyte lacuno-canalicular system (9). Mechanical loading of bone triggers interstitial fluid flow in this lacuno-canalicular system. Osteocyte dendritic processes sense the fluid flow, resulting in cellular signaling (10–12). In response to mechanical stimuli, osteocytes release nitric oxide (NO), prostaglandins (PGs), and ATP (within milliseconds), which affects many other cellular signaling pathways including interleukin-6 (IL-6), receptor activator of nuclear factor κB ligand/osteoprotegerin (RANKL/OPG), Wnt/β-catenin, and calcium signaling pathways (10, 11, 13–15). During the last 30 years various mechanisms of osteocyte mechanotransduction have been reported. Calcium oscillation in osteocytes has been shown to be a critical regulator of osteocyte mechanotransduction (16–18). Recently, mechanical loading-induced Ca^2+^ oscillation has been shown to cause the release of extracellular vesicles from osteocytes and to promote bone regeneration (19). Loading-induced Ca^2+^ oscillation in osteocytes triggers the release of downstream signaling molecules, e.g., NO (14, 20–22), prostaglandin E_2_ (PGE_2_) (23), matrix extracellular phosphoglycoprotein (MEPE), insulin-like growth factor-1 (IGF-1) (24), and β-catenin (25). Similarly, primary cilia on the osteocyte cell body as well as the dendritic processes play a regulatory role in the mechanotransduction process in osteocytes (26). Focal adhesions are macromolecular complexes consisting of multiple actin-associated proteins, such as paxillin, vinculin, connexin-43, integrins, and talin, that serve as physical linkages between a cell's cytoskeleton and the ECM. The mechanism of focal adhesion-mediated osteocyte mechanotransduction has been partly unraveled (27–30).

Osteocytes produce various signaling proteins such as sclerostin, WNT1, WNT3a, Dickkopf-related protein 1 (DKK1), phosphate regulating endopeptidase homolog X-linked (PHEX), RANKL, MEPE, fibroblast growth factor-23 (FGF23), sclerostin, and vascular endothelial growth factor (VEGF) (31–34). These proteins and growth factors not only play a crucial role in bone biology, but also in other organs such as kidney, and in fat metabolism (34, 35). Disruption of the production of these proteins by impaired osteocyte function causes bone diseases, including rare bone diseases (36–40). Osteocyte-specific release of growth factors and signaling molecules is disturbed during long-term unloading, such as occurs in astronauts during space traveling and long-term bed rest (11). Similarly, inflammatory conditions caused by various inflammatory diseases also affect osteocyte function and signaling (41, 42). Various genetic abnormality-associated rare bone diseases are related to disrupted osteocyte functions.

Wnt signaling plays a vital role in skeletal health, mainly via osteogenic differentiation of precursor cells, osteocyte viability, and osteocyte signaling to other bone cells (43, 44). Wnt/β-catenin activation in osteocytes mainly contributes to the anabolic effect in bone (45). Mechanical loading-induced early release of PGE_2_ causes rapid activation of Wnt/β-catenin signaling in osteocytes (46, 47). Wnt ligand co-receptor LRP5 is essential for osteocyte mechanotransduction and mechanical loading-induced bone formation (43, 48–50). This suggests a crucial role of osteocytic Wnt signaling in the process of mechanotransduction. The consequence of disturbed Wnt signaling in osteocytes is demonstrated by a mutation in the WNT1 gene, which causes autosomal-recessive osteogenesis imperfecta, a childhood rare bone disease (51). The osteocyte is the main source of sclerostin, a negative regulator of Wnt/β-catenin signaling. Mechanical loading reduces, while proinflammatory cytokines enhance sclerostin production in osteocytes (31, 41). Sclerostin deficiency in various rare genetic bone diseases, such as sclerosteosis and van Buchem disease, causes osteopetrosis, a high bone mass phenotype (36).

Parathyroid hormone (PTH) signaling contributes via PTH-related protein (PTHrP)-derived peptides, to the mechanical loading-induced osteocyte-mediated adaptation of bone tissue composition (52, 53). Inherited hypoparathyroidism is a rare disease that reduces bone turnover causing higher bone mineral density (BMD) and brittle bone (54). However, the osteocyte mechanotransduction-mediated bone adaptation in inherited hypoparathyroidism is still unknown. Similarly, mechanical loading upregulates insulin growth factor-1 (IGF1) expression in osteocytes, and IGF1 signaling plays an important role in the osteogenic response to mechanical loading (24, 55, 56). Moreover, IGF1 regulates PTH/PTHrP signaling in osteocytes, and bone regeneration (57–61). Osteocytic IGF1 signaling in rare bone diseases still needs to be investigated. Osteocytes produce RANKL and OPG to regulate osteoclastogenesis and osteoclast activity (6, 62). The RANKL/OPG ratio in osteocytes is upregulated by proinflammatory cytokines (31, 41, 63), but reduced by mechanical loading (64). Mechanical loading of osteocytes downregulates the expression of most proinflammatory cytokines, except IL-6. Interestingly, mechanical loading upregulates IL-6 expression in parallel with PGE_2_ production in bone cells (63, 65). However, the exact role of mechanical loading-induced osteocytic IL-6 signaling in bone biology and rare bone diseases is poorly understood. Osteocytes not only regulate osteoblast and osteoclast formation and activity, but also phosphate homeostasis and the function of vital organs in an endocrine fashion (62, 66). Osteocytes respond to PTH by inducing osteolysis that releases calcium in the bloodstream to maintain the systemic mineral homeostasis (67). During lactation, osteocytic sclerostin modulates the production of the osteoclast markers tartrate-resistant acid phosphatase (TRAP), cathepsin K, and carbonic anhydrase-2 in osteocytes to regulate the release of calcium from bone (68). Mutation of the cathepsin K encoding gene causes a rare autosomal recessive osteochondrodysplasia (69). Although cathepsin K is mainly required for osteoclastic bone resorption, osteocytes also release cathepsin K and regulate mechanotransduction (70). Osteocytes release FGF23, dentin matrix acidic phosphoprotein 1 (DMP1), PHEX, and MEPE, and act as endocrine cells to regulate phosphate metabolism (1, 71–73). Osteocytes release sclerostin to control bone mineralization via the modulation of DMP1, PHEX, MEPE, and FGF-23 expression (74, 75). The osteocyte is a critical player in chronic kidney disease-associated adverse effects on bone and heart (76). Osteocyte-derived DMP1 reduces FGF23 expression and enhances bone mineralization (35). Chronic kidney disease reduces DMP1 expression in osteocytes, while DMP1 supplementation prevents osteocyte apoptosis, lowers FGF23 expression, increases serum phosphate, and prevents the development of left ventricular hypertrophy in a chronic kidney disease mice model (35, 76). PHEX indirectly regulates FGF23, and *PHEX* gene mutation causes hypophosphatemic rickets, a rare hereditary bone disease (39). The MEPE-PHEX interaction regulates bone turnover, mineralization, and bone-renal vascularization (77). MEPE is highly expressed in human osteocytes embedded within mineralized bone (78). MEPE^−/−^ mice develop increased bone mass, hyperphosphatemia and creatinine-clearence, and transgenic overexpression of MEPE C-terminal acidic serine aspartate-rich MEPE-associated (ASARM)-motif corrects these abnormalities (79). C-terminal ASARM-motif plays a major role in regulation of bone mass and renal function in aging mice showing the association of MEPE in age-dependent osteoporosis. This unveils the endocrine function of osteocytes affecting the function of distant organs such a kidney and heart. Thus, osteocytes play a vital role in bone homeostasis, and several osteocyte-specific proteins are involved in the pathogenesis of rare bone diseases. In this review, we mainly focus on the role of disturbed development and activity of osteocytes in rare bone diseases. We will discuss the existing insights on the role of osteocytes in the pathophysiology of rare metabolic bone disorders as well as the consequences of these rare metabolic bone disorders for the development and function of osteocytes.

## Disturbed Osteocyte Function Can Cause Metabolic Bone Diseases

Many factors, including aging, osteoporosis, inflammatory diseases, and systemic diseases, disrupt osteocyte functions (2, 41, 76, 80). Aging causes 15–30% reduction in lacunar density or osteocyte numbers (81). Smaller and more round osteocyte lacunae are common in aged mice compared to young mice (82). The age-related decrease in lacunar density is accompanied by osteocyte death, hypermineralization, and micropetrosis (83). Aging also reduces the number of osteocyte dendrites and canaliculi by ~30% (80, 84). The remarkable decrease in osteocyte lacunar density, canaliculi, and dendrites number will reduce the entire osteocyte network connectivity that affects osteocyte function and bone homeostasis. Since the osteocyte lacuno-canalicular system plays a crucial role in mechanotransduction, abnormalities in this system might directly affect osteocyte mechanotransduction-mediated bone adaptation and remodeling (85). Estrogen, PTH, bisphosphonates, and muscle-derived irisin increase osteocyte survival (86–88). Postmenopausal estrogen deficiency, imbalance in PTH signaling, long-term glucocorticoid treatment, and oxidative stress caused by disuse may cause osteocyte death resulting in imbalanced bone remodeling and decreased bone mass (89). Systemic inflammatory conditions, such as periodontitis, rheumatoid arthritis, chronic kidney disease, and cancer, affect osteocyte function mainly via elevated levels of proinflammatory cytokines.

Advanced glycation end products (AGEs) are inflammatory mediators in diabetes. AGEs induce osteocyte apoptosis and upregulate osteocytic expression of IL-6 and VEGF (90, 91). Periodontitis-mediated inflammation causes sclerostin production and NF-κβ activation in alveolar osteocytes (92). Diabetic rats with periodontitis show a higher expression of sclerostin, RANKL, tumor necrosis factor-α (TNFα), and IL-1β in osteocytes, which affects osteoblast and osteoclast function (93–95). Brucella abortus infection is a common cause of osteomyelitis, which not only inhibits connexin-43 expression in osteocytes, but also induces osteocyte apoptosis and upregulates expression of inflammatory mediators RANKL, TNFα, and IL-6 in osteocytes (96). Multiple myeloma, a cancer that directly affects bone, induces osteocyte apoptosis and osteocyte-derived sclerostin and RANKL expression (97). Osteocytic sclerostin and FGF23 expression are highly upregulated in chronic kidney disease (98). In rheumatoid arthritis, a systemic inflammatory disease, elevated levels of inflammatory cytokines enhance IL-1β, TNFα, sclerostin (*SOST*), and DKK1 gene expression in osteocytes (31).

## Rare Bone Diseases and Osteocyte Function

**Figure 1:**
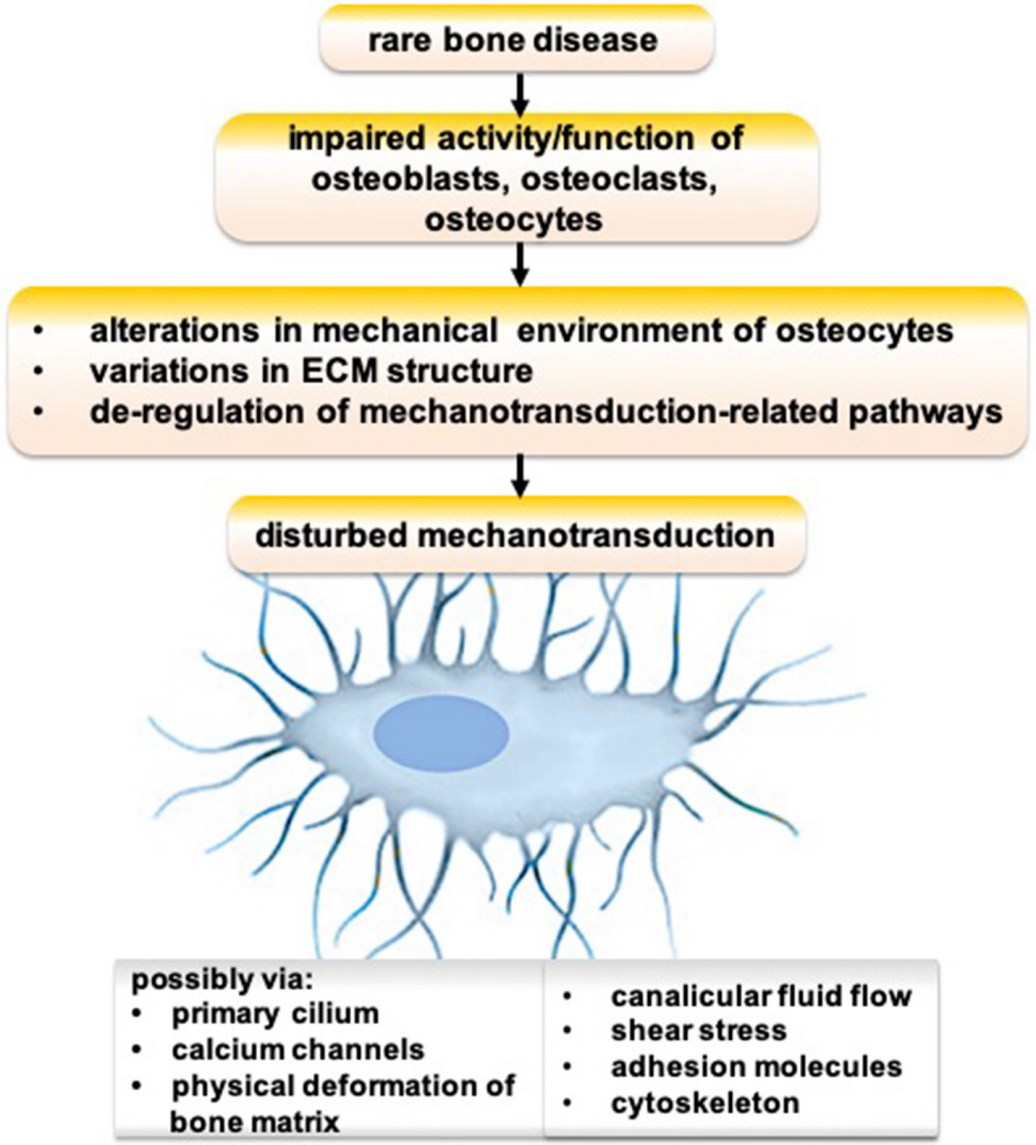
Schematic showing the possible mechanism of disrupted osteocyte functions in rare bone diseases.

Genetic defects cause various rare bone diseases such as Paget disease, fibrous dysplasia, pycnodysostosis, sclerosteosis, osteogenesis imperfecta, X-linked hypophosphatemia, and hypophosphatasia. Osteocyte functions are disturbed in many genetic defect-mediated rare bone diseases (99, 100). Possible mechanisms of disrupted osteocyte functions in rare bone diseases are depicted in Figure 1. An impaired activity/function of osteoblasts, osteoclasts, and/or osteocytes could lead to alterations in the mechanical environment of osteocytes, variations in ECM structure, and de-regulation of mechanotransduction-related pathways, resulting in disturbed mechanotransduction possibly via primary cilium, calcium channels, physical deformation of bone matrix, canalicular fluid flow, shear stress, adhesion molecules, and/or cytoskeleton.

## Sclerosteosis and van Buchem Disease

Sclerosteosis and van Buchem disease are autosomal recessive skeletal dysplasia causing deficiency of sclerostin protein and progressive skeletal growth (36). Sclerosteosis is primarily reported in the descendants of Dutch settlers from the seventeenth century in South Africa (101). Van Buchem disease is mainly found in a Dutch population in The Netherlands (102, 103). Skeletal manifestations of sclerosteosis and van Buchem disease are similar, including increased thickening of skull, jaw bones, long bones, and ribs. Gigantism, and hand abnormalities in sclerosteosis are distinguishing features between sclerosteosis and gigantism (104). *SOST*, the gene responsible for sclerosteosis and van Buchem disease, is localized on chromosome 17q12-q21, and encodes sclerostin protein. A point mutation in the *SOST* gene causes sclerosteosis, and a 52 kb deletion of the downstream gene of *SOST* causes van Buchem disease (36, 37). A study on the genetics and pathophysiology of sclerosteosis and van Buchem disease led to the discovery of sclerostin and its function that contributed to the development of an anti-sclerostin drug to treat osteoporosis (105). Mature osteoblasts produce sclerostin to some extent, but osteocytes are the primary source of sclerostin (106). Activation of Wnt/β-catenin signaling enhances osteogenic differentiation and bone formation (107). Sclerostin, a potent Wnt inhibitor, controls osteogenic differentiation of precursor cells and bone formation (108). On the other hand, Wnt inhibition causes overexpression of RANKL and deregulation of OPG resulting in osteoclastogenesis (38). Studies on rare bone diseases, sclerosteosis, and van Buchem disease, have unraveled the role of sclerostin in bone homeostasis (99). In the case of sclerostin deficiency, osteocytes become like a “snake without fang” and are unable to control new bone deposition by osteoblasts (36, 37). Sclerostin deficiency results in excessive bone formation (109), as observed in sclerosteosis and van Buchem disease. Since both sclerosteosis and van Buchem disease are genetic diseases caused by osteocytic sclerostin deficiency, the osteocyte could be the possible target cell to treat these diseases.

## Hypophosphatemic Rickets

Hypophosphatemic rickets is a hereditary disease with a prevalence of 1/20,000. *PHEX* gene mutations have been reported to cause hypophosphatemia and a hypomineralized bone phenotype (39, 40). Hypophosphatemic rickets is characterized by a generalized bone mineralization defect resulting in a decreased total volumetric bone mineral density (vBMD) at the radius and tibia, and lower cortical vBMD and cortical thickness at the radius compared to healthy adults (110). However, the exact mechanism of *PHEX* gene mutation-mediated FGF23 upregulation, hypophosphatemia, and development of rickets is still unclear. Both PHEX and FGF23 are mainly produced by osteocytes (111). One autosomal recessive hypophosphatemic rickets family carried a mutation affecting the dentin matrix protein (*DMP1*) start codon (112). DMP1 is essential for osteocyte maturation, while *DMP1* mutation leads to altered skeletal mineralization and disturbed phosphate homeostasis associated with increased FGF23 production via an effect on the function of osteocytes (112). A combination of oral phosphorous supplementation and active vitamin D analogs is the conventional therapy to counteract the consequences of excessive FGF23 in hypophosphatemic rickets (113). Anti-FGF23 antibody or gene therapy targeting *DMP1, FGF23*, or *PHEX*, could be a future direction to treat hypophosphatemic rickets. This has been demonstrated already in children with X-linked hypophosphatemia, where treatment with anti-FGF23 antibody Burosumab improved linear growth and physical function, and reduced the pain and the severity of rickets (114).

## WNT1 and PLS3 Mutation

WNT1 is a key ligand of the canonical WNT signaling pathway, which is the most important signaling pathway in bone (115). The WNT family contains a total of 19 WNT proteins, including WNT1, which are essential for fetal bone development and maintenance of postnatal bone health (38). The plastin protein family belongs to the actin bundling proteins and is ubiquitously expressed in solid tissue, including neurons in the brain, osteoblasts and osteocytes in bone, hematopoietic cells, and many cancer cell types (116). Plastin3 (PLS3) expression in mesenchymal stem cells and osteoblasts increases during osteogenic differentiation (117, 118). Missense mutation c.652T>G (p.C218G) in *WNT1*, and an X-linked form resulting from a splice mutation c.73-24T>A in PLS3 are associated with osteoporosis in children (115, 119). The role of WNT1 and PLS3 in the function of osteocytes is not yet fully understood. *WNT1* mutation affects WNT/β-catenin signaling that might affect osteocyte function, and causes an imbalance in bone homeostasis resulting in osteoporosis (51). PLS3 has been suggested to play a role in osteocyte dendrite function and mechanotransduction (120). High FGF23 expression has been reported in osteocytes of a patient with a *WNT1* mutation compared to a *PLS3* mutation (121). The expression pattern of DMP1, sclerostin, and phospo-β-catenin is similar in patients with a *WNT1* and *PLS3* mutation (121). This suggests that WNT1 and PLS3-mediated osteoporosis might have a similar mechanism of disease progression. Osteocyte-derived WNT1 is a key regulator of osteoblast function and bone homeostasis (122). Deletion of *Wnt1* in osteocytes results in low bone mass and increased fracture risk, similar as *WNT1* mutation-related osteoporosis (122). Interestingly, *Wnt1* overexpression in osteocytes stimulates bone formation by increasing the osteoblast number and activity partly via activation of mTORC1 signaling (122). Anti-sclerostin antibody robustly increases bone mass and reduces the fracture rate in *Wnt1* global knockout mice (122). These findings suggest that *WNT1* mutation-related osteoporosis is caused in part by a loss of WNT1 signaling in osteocytes, which decreases mTORC1-dependent osteoblast formation and bone regeneration. The sclerostin antibody has been suggested to be an effective treatment option for *WNT1* mutation-related osteoporosis (122). However, osteocytic mechanotransduction in patients with a *WNT1* mutation is not yet fully understood. Microgravity, or unloading, decreases WNT3a, WNT5a, DKK1, cyclinD1, LEF-1, and CX43, but increases *WNT1* and *SOST* expression in osteocytes (11, 123). Microgravity dramatically reduces the number of F-actin filaments in osteocytes (123). This suggests a role for WNT1 in the formation of the osteocyte cytoskeleton and in osteocyte mechanosensitivity. *PLS3* mutation or deficiency causes low bone mass, possibly via hyperactivity of osteoclasts. PLS3-deficient mice show no effect in trabecular bone, but cortical bone mass is highly reduced (124). Normal osteocyte morphology is observed in PLS3-deficient mice (125). Bone marrow stem cells from PLS3-deficient mice show compromised osteogenic differentiation with reduced expression of osteocalcin, Wnt16, and Sfrp4 mRNA (125). This indicates a role of PLS3 in bone regeneration via osteoblast differentiation and function (125). A lack of PLS3 has been shown to decrease the expression of NFkB repressing factor, thereby augmenting Nfatc1 transcription and osteoclastogenesis, indicating osteoclast-mediated bone loss in PLS3-deficient mice (124). The actin cytoskeleton and focal adhesions play an important role in osteocyte mechanotransduction. Since the plastin protein family belongs to the actin bundling protein, plastin might have a direct or focal adhesion-mediated indirect effect on osteocyte mechanotransduction. However, the role of PLS3 in osteocyte functions, such as mechanotransduction, osteocyte-to-osteoblast communication, and osteocyte-to-osteoclast signaling, and its cellular and molecular influence on bone remodeling has not been investigated yet.

## Osteogenesis Imperfecta

Osteogenesis imperfecta is mainly an autosomal dominant disease of connective tissue that lowers bone mass and causes fracture. Very few cases of recessive and X-chromosome-linked forms of osteogenesis imperfecta have been reported so far. Osteogenesis imperfecta is one of the most common bone fragility disorders with an incidence of about 1/15–20,000 (126). It is a brittle bone disease directly related to abnormalities of type I collagen primary posttranslational modification, folding, structure, strength, and quantity (127). Mutations in the *COL1A1* or *COL1A2* gene, encoding the α1(I) or α2(I) chain of type I collagen, are associated with ~85% of osteogenesis imperfecta cases (128). Mutation-mediated alteration in processing, structure, and secretion of type I collagen, as well as ER stress causes a subclinical to lethal skeletal phenotype. Loss-of-function mutations in WNT1 lead to moderately severe and progressive forms of osteogenesis imperfecta (119, 129). Since osteocytes are embedded in the bone ECM, ECM-to-osteocyte interaction plays a vital role in bone homeostasis. The effect of deregulated collagen matrix-to-osteocyte interaction in osteogenesis imperfecta could influence the severity of bone fragility. However, the role of osteocytes in osteogenesis imperfecta disease progression has rarely been investigated yet. Future studies focusing on the role of the collagen matrix-to-osteocyte interaction in osteocytes function, including mechanotransduction, and osteoblast-to-osteoclast communication could guide in the development of new therapeutic targets to treat osteogenesis imperfecta.

## Pycnodysostosis

Pycnodysostosis (OMIM 265800) is a rare autosomal recessive osteochondrodysplasia with a prevalence rate of 1–1.7/million and without gender specificity (130). Pycnodysostosis is characterized by a short stature with increased bone mineral density and an increased bone fragility phenotype (105, 131). Cortical and trabecular osteosclerosis with increased cortical width and high bone mineral density is observed in patients with pycnodysostosis (11, 12). Gelb et al. reported mutation of the gene encoding cathepsin K in chromosome 1q21 in patients with pycnodysostosis (69). Cathepsin K degrades bone matrix proteins, including collagen type I, and is therefore essential for osteoclastic bone resorption (132). A study on the genetics and pathophysiology of pycnodysostosis revealed the role of cathepsin K in osteoclast activity that led to the development of cathepsin K inhibitors to treat osteoporosis by inhibiting osteoclastic bone resorption (105). Unfortunately cathepsin K inhibitors did not lead to new osteoporosis medication because of serious side effects (stroke). In pycnodysostosis the number of osteoclasts is not affected, but bone resorption is highly reduced (133). Osteoclastic bone resorption is essential for bone homeostasis, as old and cracked bone is removed as well as the fibrous extracellular matrix that provides the signal to osteoblasts to deposit new bone and increase bone strength. Cathepsin K is also produced by osteoblasts and osteocytes (70, 134). Osteocytic cathepsin K is responsible for lactation-induced bone loss (135). Mechanical loading increases cathepsin K expression in cortical bone of wild type mice (70). Globally knocking out of cathepsin K enhances mechanotransduction signals resulting in cortical bone formation (70). Cathepsin K regulates bone remodeling not only by enhancing osteoclast activity, but also by inhibiting osteogenic differentiation via modulation of Wnt signaling (70). Cathepsin K deficiency in osteoclasts increases sphingosine kinase 1 (Sphk1) that catalyzes the phosphorylation of sphingosine to sphingosine-1-phosphate (S1P) (136, 137). S1P promotes osteoblast differentiation, bone regeneration (136), and osteocytic mechanotransduction (138). New research approaches reducing the mechanosensitivity of osteocytes by inhibiting S1P could be important to develop therapeutics for the treatment of cathepsin K deficiency-mediated high bone mass phenotype.

Cathepsin K regulates bone remodeling and cortical bone formation by degrading periostin (139). Periostin is mainly expressed in the periosteum and in osteocytes, and enhances bone formation via activation of Wnt signaling (70). Bonnet et al. nicely depicted the role of osteoblastic and osteocytic periostin in cathepsin K-mediated bone modeling and remodeling (70). Osteocyte-mediated periostin could be a possible target in pycnodystostosis.

## Analysis of Osteocyte Function

Multiple approaches have been developed to analyze osteocyte morphology (80). Confocal laser scanning electron microscopy (CLSM) (140), scanning electron microscopy (SEM) (141), ultra-high voltage electron microscopy, tomography on silver stained bone sections (117, 142), and SEM of acid-etching technique of non-decalcified bone samples (143) have been developed to visualize osteocyte density, morphology, and osteocyte lacuno-canalicular network in bone biopsies from patients. Van Hove and colleagues nicely show differences in osteocyte morphology in patients with osteoarthritis, osteopenia, and osteopetrosis using CLSM (144). Schneider and colleagues developed serial focused ion beam/SEM imaging for quantitative 3D-assessment of the osteocyte lacuno-canalicular network (145). Micropetrotic lacunae, as seen in old age, in cortical and trabecular bone can be visualized by transmission electron microscopy (TEM) and SEM (81). High power backscattered SEM images of a bone tissue section visualizes the mineralized micropetrotic lacunae (146). Osteocyte-specific expression of proteins such as sclerostin, IL-1β, TNFα, DKK1, DMP1, and FGF23 is altered in different disease conditions. Immunohistochemistry using specific antibodies easily visualizes the expression pattern in bone sections (33, 121, 147). Serum sclerostin is a key marker of osteocyte function in different disease conditions (148, 149). Serum sclerostin levels are upregulated in osteoporosis and downregulated in high bone mass conditions (150). Enzyme-linked immune assays and automated chemiluminescent assays have been developed and validated for high precision analysis of serum sclerostin (151, 152). Spinal cord injury causes patient immobilization and bed rest that mimics unloading conditions. Serum of patients with spinal cord injury contains increased periostin and decreased sclerostin levels (153). Since sclerostin and periostin are mainly secreted by osteocytes, these proteins could possibly be used as serum markers to analyze osteocyte function in different diseases.

Osteocyte mechanotransduction alters in different disease conditions, such as aging, osteoporosis, and inflammatory diseases (82, 154–157). Various *in vitro* and *ex vivo* methods have been developed to analyze osteocyte functions (158). However, most of these methods are invasive and difficult to perform routinely in clinical setting. Non-invasive bone loading methods are available to analyze osteocyte functions in murine models (59, 159, 160). Future research is recommended to develop non-invasive approaches to analyze osteocyte mechanotransduction *in vivo*.

Recently, extracellular vesicles and exosomes are regarded as the key cargo-carrying organelles affecting the local and systemic cellular activities. Exosomes are released from living cells and carry miRNAs, circular RNAs, mRNAs, and various proteins from one cell to other cells. Osteocyte-derived exosomes detected in the circulation are enriched with osteocyte-specific miRNAs (161). A possible role of extracellular vesicles and exosomes in bone biology has been presented nicely in a recent review from Tao and Guo (162). Mechanically loaded osteocytes release exosomes with bone regenerating potential, via Ca^2+^ oscillation (19). Proteomic analysis of exosomes from cortical bone osteocytes provide a clear picture of osteocyte function in different disease conditions, including rare bone diseases (32). The osteocyte transcriptome is extensively deregulated in a mouse model of osteogenesis imperfecta (163). Transcriptome and proteomic analysis in osteocytic exosomes could unravel the role of exosomes in the pathophysiology of rare bone diseases. Recent advancements in RNA sequencing, functional analysis tools, and bioinformatic tools reveal a role of non-coding RNAs such as miRNAs, circular RNAs, piRNAs, and lncRNAs in various cellular signaling and biological activities including development and diseases (164–168). Various mRNAs and their translated proteins play a role in osteocyte function (36, 56). Only few studies address the role of non-coding RNAs in osteocyte function (161, 169, 170). Disruption of the Cx43/miR21 pathway results in osteocyte apoptosis and increases osteocyte-mediated osteoclastogenesis in old-age subjects (170). miR-29b-3p regulates osteogenic differentiation of precursor cells via modulating IGF1 secretion in mechanically loaded osteocytes (169). The role of circular RNAs, piRNAs, lncRNAs, and other miRNAs on osteocyte functions in physiological and disease conditions is poorly understood. The differential expression pattern of non-coding RNAs in osteocytes during rare bone diseases has not been investigated yet. Altered expression pattern of non-coding RNAs in osteocytes during rare bone diseases could play role in disease development and pathophysiology. We believe that this research direction could guide the development of new targets and techniques to analyze the function of osteocytes in patients.

## Therapies to Improve Osteocyte Function

Intermittent PTH therapy enhances bone regeneration and bone mineral density (171). PTH signaling affects the function of osteoblasts, osteoclasts, and osteocytes. Intermittent PTH treatment enhances the commitment of precursor cells to an osteogenic fate (172). PTH signaling in osteocytes regulates sclerostin expression and controls osteocyte-mediated osteoblastogenesis (58, 87, 173, 174). PTH treatment (teriparatide, PTH1-34) in osteogenesis imperfecta increases bone mineral density and vertebral strength (175, 176). PTH inhibits Notch signaling in osteoblasts and osteocytes, which might exert the anabolic effect on bone (177).

Studies on sclerostin deficiency-related high bone mass phenotype illustrate the role of sclerostin in bone biology guiding the development of anti-sclerostin bone anabolic agents. Anti-sclerostin monoclonal antibody has the potency to treat diseases with low bone mass phenotype, including osteoporosis (178, 179). There is increasing evidence suggesting a role of sclerostin in myeloma bone diseases and breast cancer bone metastasis-mediated complications (149, 180). In the bone niche, sclerostin is mainly produced by mature osteoblasts and osteocytes (181). Interestingly, multiple myeloma cells and breast cancer cells also produce sclerostin that might have a catabolic effect on bone (180, 181). Furthermore, cancer metastasis-induced inflammation upregulates osteocytic sclerostin that inhibits osteoblast function (181). Therefore, sclerostin monoclonal antibody could be beneficial to reduce myeloma and breast cancer-mediated complications in bone (182–184). Sclerostin antibody romosozumab clears a phase III trial with satisfactory outcomes and already got approval for osteoporosis treatment (185). This sclerostin antibody has shown promising potential to treat osteogenesis imperfecta (127, 186, 187). Therefore, romosozumab might be beneficial to treat rare bone disease patients with low bone mass phenotypes, such as osteogenesis imperfecta, Wnt1 mutation, and PLS3 mutation.

DKK1 is another potent Wnt inhibitor, that is also mainly produced by osteocytes in bone. Similar to sclerostin, DKK1 is also produced by breast, prostate, and multiple myeloma cancer cells (188–190). Increased levels of DKK1 in various cancers cause osteolytic bone disease and inhibit osteoblast function (188, 189). DKK1 is an osteocyte-specific target to treat osteoporosis and other low bone mass diseases (191). In systemic inflammation, the neutralization of DKK1 reduces sclerostin expression and protects systemic bone loss (192). Monoclonal antibodies against DKK1 showed DKK1 inhibitory potential *in vitro* and increased bone mass *in vivo* (192). Moreover, a bispecific antibody targeting both sclerostin and DKK1 shows higher efficiency on bone formation and fracture repair (193). Phase I and phase II clinical trials have been performed to test the efficacy of anti-DKK1 antibodies on myeloma and myeloma-induced skeletal events (194, 195).

Studies on the role of osteocytic RANKL in bone homeostasis have led to the development of an anti-RANKL monoclonal antibody to treat common metabolic bone diseases, including osteoporosis (196, 197). During the last 10 years, the use of denosumab proved to be satisfactory with rare adverse effects (198). An imbalance in RANK-RANKL-OPG signaling is also observed in many rare bone diseases such as Juvenile Paget disease, fibrous dysplasia, Hajdu Cheney syndrome, and Langerhans cell histiocytosis (199). Therefore, denosumab has also been used off-label in rare metabolic bone diseases, including Paget's disease, osteogenesis imperfecta, and aneurysmal bone cysts (200). Bisphosphonate treatment prevents bone loss and fractures caused by rare bone disease-mediated osteogenesis imperfecta (201–203). Physical therapy/rehabilitation regimes in children with osteogenesis imperfecta improved mobility and bone mineral density, and thereby prevented fractures (175). Most treatment approaches for rare bone diseases directly act on osteoblast or osteoclast activity, and are symptomatic treatments.

**Figure 2:**
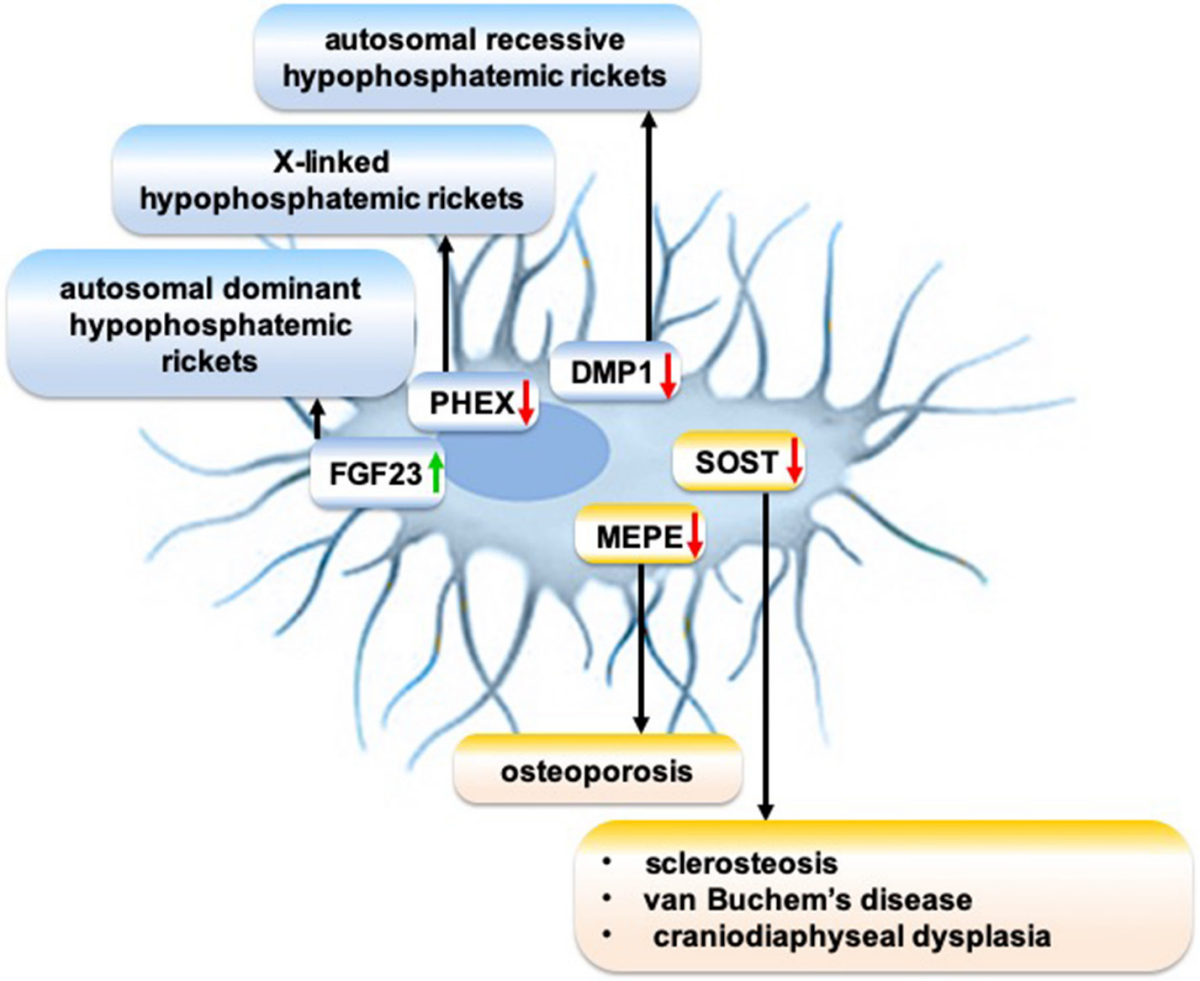
Schematic showing the role of disrupted expression of osteocytic proteins on the pathophysiology of rare bone diseases. Green arrow: Gain-of-function mutation; red arrow: Loss-of-function mutation.

The meticulous research on the molecular mechanism of osteocytic sclerostin on bone remodeling led to the development of anti-sclerostin antibodies to treat osteoporosis and other skeletal disorders demanding an increase in bone mass. Anti-sclerostin antibody primarily targets bone-lining cells, rather than the osteocytes imbedded in bone matrix (204). Anti-sclerostin antibody activates selected canonical Wnt target genes in a mature osteoblast subpopulation and increases bone formation (204). Sclerostin monoclonal antibody romosozumab treatment significantly increases bone mineral density in postmenopausal women with low bone mineral density and reduces fracture risk in postmenopausal women with osteoporosis (205). However, adverse side effects of a loss of sclerostin are osteoarthritis (206), TNF-dependent inflammatory joint destruction (207), negative effect on B cells (208), and risk of cardiac failure (205), which should be carefully evaluated before romosozumab treatment is considered. Although research on the cellular and molecular mechanisms of sclerosteosis and van Buchem disease guided the development of anti-sclerostin antibody to treat osteoporosis, an osteocyte function-targeted therapy for sclerosteosis and van Buchem disease has not yet been developed. Genetic disorders disrupt the expression of osteocytic proteins that play a role in the pathophysiology of various rare bone diseases (Figure 2). Since osteocyte functions play a crucial role in bone homeostasis, and since these functions are disrupted in many rare bone diseases, a better understanding of the molecular mechanisms of disrupted osteocyte functions in rare bone diseases may guide to discover novel targets to treat these rare bone diseases.

## Conclusions

Genetic and pathophysiological research on three rare bone diseases, i.e., sclerosteosis, pycnodysostosis, and van Buchem disease, provided new effective interventions to treat osteoporosis. The current available therapeutic approaches for rare bone diseases are symptomatic and mainly target osteoblast and osteoclast formation and activity. Since osteocytes play a vital role in bone homeostasis, and because their function is disrupted in many rare bone diseases, it would be wise to focus on unraveling the osteocyte-specific targets to treat rare bone diseases. The role of coding RNAs (mRNAs) in osteocyte function during pathophysiological conditions has been widely investigated. Non-coding RNAs (piRNAs, circRNAs, lncRNAs, shRNAs, etc.) represent 97% of the total RNA in the cell, and recent technological advances have unveiled a crucial role of non-coding RNAs in various biological processes including bone homeostasis. Therefore, meticulous research focusing on the role of non-coding RNAs in osteocyte functions under physiological conditions and in various bone diseases including rare bone diseases could be the future research direction. The results of this research could provide clues for the discovery of novel osteocyte-specific targets to treat rare bone diseases.

## Author Contributions

All authors listed have made a substantial, direct and intellectual contribution to the work, and approved it for publication.

## Conflict of Interest

The authors declare that the research was conducted in the absence of any commercial or financial relationships that could be construed as a potential conflict of interest.
